# A Cardiologists’ Survey on the Use of Anticoagulants and Antiplatelets in Patients With Atrial Fibrillation and Acute Coronary Syndrome or Those Undergoing Percutaneous Coronary Intervention in India

**DOI:** 10.7759/cureus.35220

**Published:** 2023-02-20

**Authors:** Namrata Kulkarni, Santosh Taur, Jaspreet Kaur, Ravishankar Akolekar, Swetha ES

**Affiliations:** 1 Department of Medical Affairs, Pfizer Ltd., Mumbai, IND

**Keywords:** vitamin k antagonists, ticagrelor, prasugrel, clopidogrel, aspirin, apixaban

## Abstract

Purpose: The management of patients with atrial fibrillation (AF) and acute coronary syndrome (ACS) or undergoing percutaneous coronary intervention (PCI) requires appropriate antithrombotic regimens for stroke prevention and in-stent thrombosis. Current practice recommendations are largely based on consensus options as there is limited evidence from randomized clinical trials. Hence, by surveying a group of cardiologists across India, we sought to better understand the current practice patterns of using oral anticoagulants (vitamin K antagonist, VKA or non-vitamin K antagonist oral anticoagulant, NOAC) and antiplatelet therapy in those patients in India.

Methods: A cross-sectional questionnaire-based survey was conducted across India to better understand the clinical practices in AF management.

Results: A total of 151 cardiologists participated in this survey. The most commonly prescribed combination therapy in patients with AF and ACS/undergoing PCI was triple therapy (NOAC + dual antiplatelet [aspirin and P2Y12 inhibitor]) (54.30%) followed by NOAC + single antiplatelet (33.11%). Only 11.26% of cardiologists prescribed VKA + dual antiplatelet therapy. Among anticoagulants, cardiologists prescribed NOACs to 66.11% of patients and VKAs to 25.54% of patients. Among P2Y12 inhibitors, ticagrelor (50.99%) and clopidogrel (47.02%) were the most preferred medication. The physician reported patient adherence rates to NOACs were higher compared to VKAs. Around 41.06% of cardiologists reportedly changed antiplatelet therapy for patients from dual antiplatelet to single antiplatelet therapy in three months; 36.42%, in one month; and 19.21% in six months after PCI. Around 61.59% of cardiologists stopped prescribing antiplatelet therapy for patients by one year.

Conclusion: Our survey demonstrated that the majority of cardiologists used triple therapy (NOAC + dual antiplatelet), followed by NOAC + single antiplatelet for managing patients with AF and ACS or undergoing PCI in line with the available guidelines.

## Introduction

Atrial fibrillation (AF) is a global health burden and increases the risk for thromboembolic complications including stroke and other cardiovascular (CV) events [1]. Acute coronary syndrome (ACS) commonly occurs in patients with AF and requires oral anticoagulants (OAC) for the prevention of stroke, in-stent thrombosis, or recurrent cardiac events [2].

Choosing the best anti-thrombotic therapy for these patients is quite challenging as it is difficult to balance thrombosis prevention and the risk of bleeding [3]. Currently, OAC such as vitamin K antagonists (VKAs) and non-VKA OAC (NOAC) are indicated to prevent AF-related ischemic strokes and systemic embolism. However, they are reportedly ineffective in preventing stent thrombosis and are not indicated for secondary prevention after ACS [4-7]. For percutaneous coronary intervention (PCI) or ACS, dual antiplatelet therapy (DAPT) with aspirin and P2Y12 receptor inhibitor (clopidogrel, prasugrel, or ticagrelor) is the gold standard and has proven to reduce the incidence of ischemic events or stent thrombosis but is less effective in reducing cardioembolic stroke [7-10]. To reduce both stroke and ischemic events, triple therapy with OAC and DAPT has been used in the past decade [10]. However, studies have shown that these regimens increase the risk of bleeding [11-13]. Thus, an antithrombotic regimen with an acceptable benefit-risk profile would be valuable in treating patients with AF and associated ACS or PCI.

Several randomized clinical trials have been conducted to evaluate the safety and efficacy of NOACs versus VKA as a combination with single or dual antiplatelet agents in patients with AF and ACS or undergoing PCI [7,14-16]. The RE-DUAL PCI trial demonstrated a lower risk of bleeding among those receiving dual therapy with dabigatran and a P2Y12 inhibitor compared to those receiving triple therapy with warfarin (P2Y12 inhibitor) and aspirin [16]. POINEER AF-PCI trial showed that low-dose rivaroxaban with a DAPT for 6-12 months was associated with low risk of clinically significant bleeding than the therapy with VKA plus DAPT [15].

The AUGUSTUS trial data support use of apixaban (NOAC) and a P2Y12 inhibitor without aspirin during the first six months for most patients owing to an almost twofold increase in bleeding with aspirin use [7]. However, despite the evidence provided by clinical trials and clinical practice guideline recommendations, treatment decisions and the choice and dosage of a particular drug can depend on the patient's clinical profile, cardiologists, and organizational factors [17].

TREAT-RISK, a major global transnational survey of AF treatment patterns reported that appropriate patients with AF are prescribed anticoagulants in the United States (82% of eligible patients on average) and its prescription is the lowest in China (58.1%) [18]. It was observed that minimizing bleeding risk was most important to Indian (7%) and Chinese (7%) healthcare providers [18]. Cardiologists in India (47%) and China (43%) were also least likely to prescribe an anticoagulant for patients over the age of 75 compared to cardiologists in the United Kingdom (92%) and the United States (88%) who were far more likely to frequently anticoagulated patients over age 75 [18].

Hence, by surveying a group of cardiologists across India, we sought to better understand the current practice patterns of using anticoagulants and antiplatelet therapy in an Indian patient with AF and ACS or those undergoing PCI and to identify prescription regimens and physician-reported patient adherence to NOACs and VKAs in clinical practice. These data reveal the current attitude of cardiologists towards prescribing anticoagulants and antiplatelet drugs.

## Materials and methods

This study was a cross-sectional questionnaire-based survey of cardiologists across India to better understand the clinical practices in AF management for 6 months in the year 2020. Cardiologists with at least 15 years of experience in the field were invited to participate in the survey. We obtained informed consent from all participating cardiologists before capturing their responses in the survey. The survey focused on the use of OAC and antiplatelets in the Indian patient population with AF and ACS or undergoing PCI. We intended to get insights into the prevailing practice of using an antithrombotic in Indian patients. The questionnaire was validated by ten cardiologists representing different geographies of the country. The questionnaire was characterized by categories on the prevalence of AF, AF, and ACS undergoing PCI and medical treatment, and anticoagulant and antiplatelet usage patterns. The entire survey was conducted using emails, short message service (SMS), and telephonic calls, the survey link was shared with the Cardiologists through email after they have consented to participate in the survey. As the study did not involve the collection or analysis of human data, ethics committee approval was not deemed necessary.

Statistical analyses

Continuous variables were summarized using descriptive statistics including the number of observations, mean, standard deviation (SD), median, and range. Categorical data were summarized as numbers and percentages. Data were summarized/analyzed per the availability of the observed dataset. All statistical analyses were performed using SAS version 9.4 (SAS Institute Inc., Cary, NC, USA).

Since this was a descriptive survey, a former effect size calculation was not required. However, an attempt was made to enroll an adequate number of cardiologists (150) to achieve an appropriate effect size. Since this is a survey about the experiences and preferences of physicians in their practice, and no human clinical data was collected, ethics committee approval was not deemed to be necessary by the authors.

## Results

The response rate was (54%, 151/280) in this survey.

Geographical distribution of responses

Upon analyzing the geographical distribution of survey responses (Table 1), the response to the survey was received more from the southern region (48.34%) followed by the northern (19.21%), western (16.56%) and the eastern (5.96%) regions.

**Table 1:** Prevalence of atrial fibrillation, acute coronary syndrome and its management in routine clinical practice of the cardiologists participating in the survey Abbreviations: AF: atrial fibrillation; ACS: acute coronary syndrome; PCI: percutaneous coronary intervention

Variables	N=151 Median (minimum, maximum)/n (%)
Geographical distribution of responses	
East	9 (5.96)
West	25 (16.56)
North	29 (19.21)
South	73 (48.34)
Not specified	15 (9.93)
Prevalence of AF in clinical practice	12 (1, 90)
Prevalence of AF & ACS or undergoing PCI in clinical practice	5 (1, 80)
Proportion of patients with ACS undergoing below treatments in clinical practice	
Medical management	20 (0, 90)
PCI	80 (2, 100)

Prevalence of AF and ACS in clinical practice

A total of 151 cardiologists participated in this survey from different regions across India. The median prevalence of AF, and AF with ACS or those undergoing PCI in clinical practice was reported as 12% and 5%, respectively. Around median of 20% patients with ACS underwent medical management and 80% were reported to undergo PCI (Table 1).

Usage pattern of antiplatelets and anticoagulants

**Figure 1:**
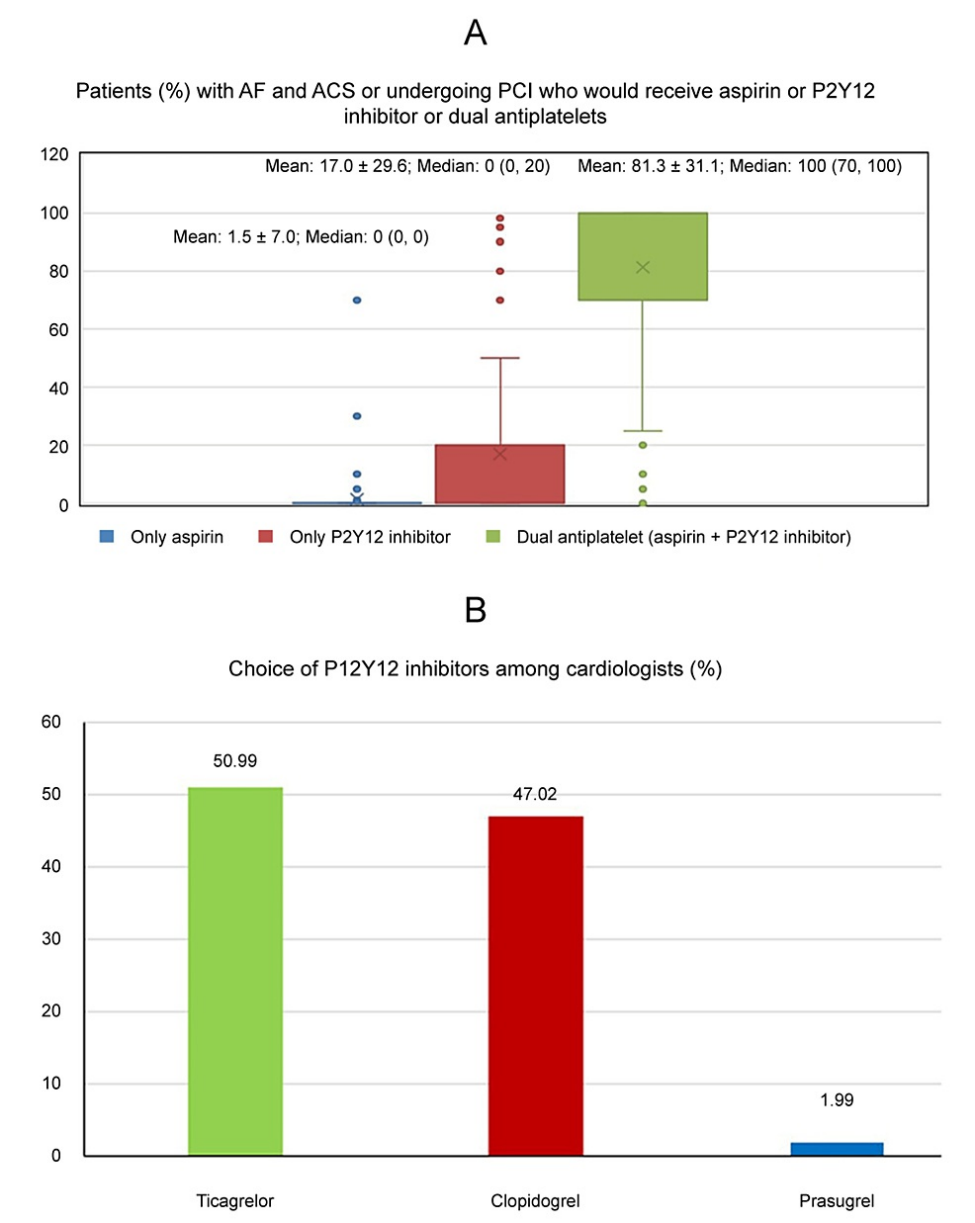
(A) Patients (%) with AF and ACS or undergoing PCI who would receive aspirin or P2Y12 inhibitor or dual antiplatelets. (B) Choice of P2Y12 inhibitors among cardiologists. Abbreviations: AF: atrial fibrillation; ACS: acute coronary syndrome; PCI: percutaneous coronary intervention

As shown in Figure 1A, cardiologists most commonly selected DAPT comprising aspirin combined with P2Y12 inhibitor (median {min., max.}: 100% {0,100}) followed by P2Y12 inhibitor alone (median {min., max.}: 0% {0, 100}). Aspirin was preferred less often (median {min., max.}: 0 {0, 70}). Among P2Y12 inhibitors (Figure 1B), ticagrelor (50.99%) and clopidogrel (47.02%) were the most preferred medication by cardiologists while prasugrel was the least preferred (1.99%).

**Figure 2:**
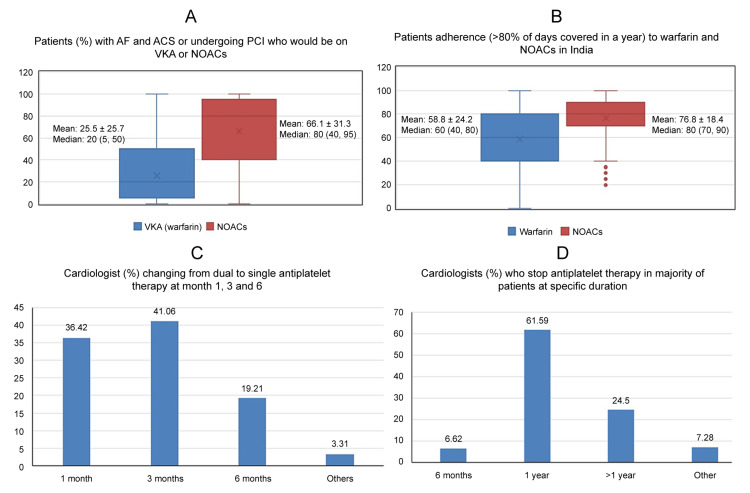
(A) Patients (%) with AF and ACS or undergoing PCI who would be on VKA or NOACs. (B) Patients adherence (>80% of days covered in a year) to warfarin and NOACs in India. (C) Cardiologist (%) changing from dual to single antiplatelet therapy at month 1, 3, and 6. (D) Cardiologist (%) who stop antiplatelet therapy in majority of patients at specific duration. Abbreviations: AF: atrial fibrillation; ACS: acute coronary syndrome; PCI: percutaneous coronary intervention; VKA: vitamin K antagonists; NOACs: non-vitamin K antagonist oral anticoagulants

Among anticoagulants, NOACs was most commonly prescribed by the cardiologists than VKA (median {min, max}: 80% {0, 100} vs 20% {0, 100}) (Figure 2A). Physician reported patient adherence rates to NOACs was higher compared to VKAs ((median {min, max}: 80% {20, 100}) vs 60% {0,100}) (Figure 2B).

**Figure 3:**
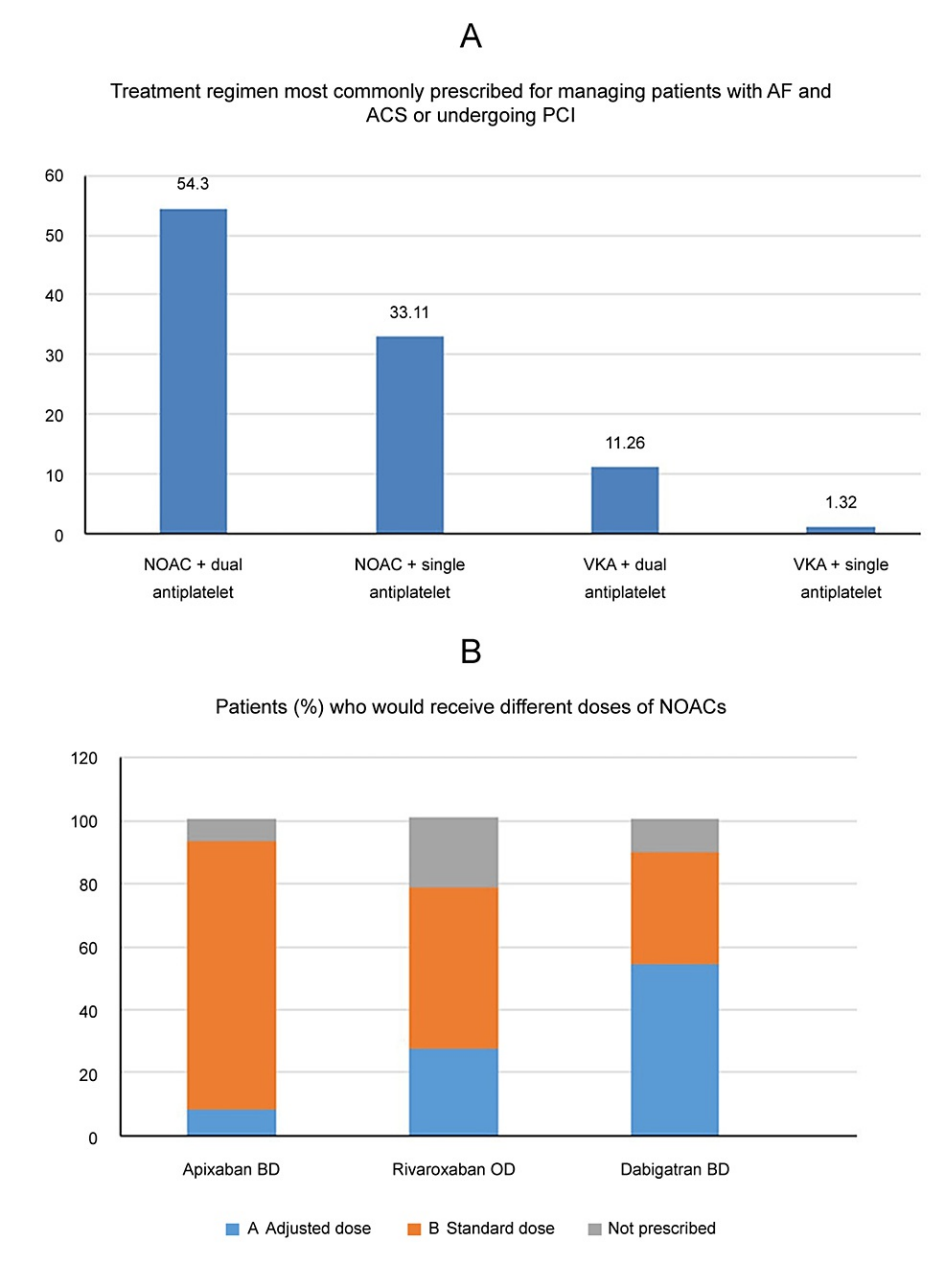
(A) Treatment regimen most commonly prescribed for managing patients with AF and ACS or undergoing PCI. (B) Patients (%) who would receive different doses of NOACs. Abbreviations: AF: atrial fibrillation; ACS: acute coronary syndrome; PCI: percutaneous coronary intervention; VKA: vitamin K antagonists; NOACs: non-vitamin K antagonist oral anticoagulants; OD: once a day; BD: twice a day

The most commonly prescribed combination therapy for managing patients with AF and ACS or those undergoing PCI was triple therapy comprising NOAC combined with dual antiplatelet (54.30%) followed by NOAC with single antiplatelet (33.11%). Only 11.26% of cardiologists prescribed VKA with DAPT (Figure 3A).

Around 41.06% of cardiologists reportedly changed antiplatelet therapy for patients from dual antiplatelet to single antiplatelet therapy in three months; 36.42%, in one month; and 19.21% in six months (Figure 2C). Around 61.59% of cardiologists stopped prescribing antiplatelet therapy for patients by one year and 24.50% stopped after one year (Figure 2D).

Prescribed dose of NOACs in clinical practice

Among NOACs, 5 mg BD (twice daily) dose of apixaban (83.22%), 20 mg OD (once daily) of rivaroxaban (42.28%) and 110 mg BD of dabigatran (51.33%) were the most commonly prescribed doses than other doses (Figure 3B).

Pattern of antiplatelet therapy prescribed with the most commonly prescribed dose of Apixaban

With the most commonly prescribed dose of apixaban (5 mg OD), if we consider the median estimates, P2Y12 inhibitor was prescribed no patients, and DAPT (Aspirin + P2Y12 inhibitor) was prescribed to 100% (0,100) of patients. Ticagrelor (50.0%) and clopidogrel (49.19%) were equally preferred by cardiologists along with apixaban 5 mg OD (Table 2).

**Table 2:** Pattern of antiplatelet therapy prescribed with the most commonly prescribed dose of Apixaban (5 mg BD)

	Median (minimum, maximum)/n (%)
Proportion of patients who were prescribed only P2Y12	0 (0, 100)
Proportion of patients who are prescribed dual antiplatelet therapy (Aspirin+P2Y12 inhibitor)	100 (0, 100)
Choice of P2Y12 inhibitor, N (%)	
Ticagrelor	62 (50.0)
Clopidogrel	61 (49.19)
Prasugrel	1 (0.81)

## Discussion

The present survey assessed the pattern of anticoagulant use in patients with AF and ACS or those undergoing PCI. The major findings of this survey reported that the majority of the cardiologists used triple therapy, and predominantly used NOAC + dual antiplatelets (aspirin and P2Y12 inhibitor) followed by NOAC + single antiplatelet for managing patients with AF and ACS or those undergoing PCI. This was in accordance with the recommendations from the current clinical practice guidelines for managing these patients [19]. The VKA + DAPT was prescribed by only 11.26% of cardiologists.

The physician reported patients’ adherence to NOACs was higher compared to VKAs in our survey. Several previous studies reported that the persistence and adherence of patients to NOACs were higher compared to VKAs due to the safety, simplified dosage regimen, and predictable dose-related anticoagulant effect enabling fixed dosing without the need for routine laboratory monitoring [20-22]. The choice for P2Y12 inhibitors was heterogeneous as cardiologists preferred both ticagrelor and clopidogrel however, prasugrel was the least preferred, probably due to the risk associated with bleeding. A recent meta-analysis showed that thrombolysis in myocardial infarction (TIMI) major and minor bleeding events were increased with the usage of prasugrel compared to clopidogrel and ticagrelor in patients with ACS [23]. The NOAC practical guide [24], EHRA consensus [25] and the ESC's DAPT-focused update [26], and the AF guideline [27] discourage using prasugrel or ticagrelor as components of a triple therapy regimen.

In our survey, around 41.06% of cardiologists were reported to change antiplatelet therapy for patients from dual antiplatelet to single antiplatelet therapy in three months, 36.42% in one month, and 19.21% in six months after PCI. Most cardiologists stopped prescribing antiplatelet therapy for patients within one year (61.59%), which is in line with the clinical recommendations based on the clinical scenario characteristics. The AAC/AHA and ESC guidelines recommend using antiplatelet therapy varying from 1 month to an extended duration of more than 12 months depending on the clinical scenario and susceptibility to ischemia, bleeding, or both in patients with AF and ACS or those undergoing PCI [19,26]. A recent North American consensus statement recommends NOACs as the oral anticoagulation of choice, and a dual therapy with NOAC and a P2Y12 inhibitor for six to 12 months is recommended based on the ischemic and bleeding risk of the patients [28].

Among anticoagulants, prescriptions for NOACs were much higher compared to VKAs as recommended in the guidelines [19]. NOACs have multiple pharmacological advantages over VKAs such as rapid onset/offset of action, few food and drug interactions, predictable pharmacokinetics and they do not require regular monitoring for coagulation [29,30]. A meta-analysis of 12 studies on the efficacy and safety of NOACs over warfarin demonstrated that NOACs are superior to the latter in preventing a composite of stroke and systemic embolism in patients with AF [31]. Moreover, NOACs appear to offer advantages over VKAs, by providing the potential for less bleeding [32,33]. These advantages might have increased their preference among Indian cardiologists.

AUGUSTUS study and ARISTOTLE study demonstrated that apixaban exerted comparable and favorable effects on preventing stroke, systemic embolism, and mortality and caused less major bleeding than warfarin irrespective of aspirin use [7,34]. Moreover, among NOACs, apixaban demonstrated lower rates of both ischemic stroke or systemic embolism and bleeding compared with rivaroxaban in patients with AF [35,36]. In the AUGUSTUS trial, the use of apixaban at a dose of 5 mg BD was shown to be an effective regimen that is superior to VKAs [7]. Thus, 5 mg BD of apixaban (83.22%) with DAPT was the most commonly prescribed dose by cardiologists in our survey.

Our study is limited by a descriptive survey and the responses are from physician reported which may not provide a true representation of patients’ responses.

## Conclusions

Our survey demonstrated that the majority of cardiologists used triple therapy (NOAC + dual antiplatelet [aspirin and P2Y12 inhibitor]), followed by NOAC + single antiplatelet for managing patients with AF and ACS or undergoing PCI in line with the available guidelines. NOACs were the preferred anticoagulant among responders in this study. The physician reported patient adherence rates to NOACs were higher compared to VKAs. 5 mg BD of apixaban with DAPT was the most commonly prescribed dose by cardiologists in our survey. Among P2Y12 inhibitors, ticagrelor and clopidogrel were the most preferred medication. The majority of cardiologists reportedly changed antiplatelet therapy for patients from dual antiplatelet to single antiplatelet therapy in three months, followed by cardiologists in one month, and a very small proportion of cardiologists in six months. A maximum number of cardiologists stopped prescribing antiplatelet therapy for patients by one year.

## References

[1] Nesheiwat ZGA, Jagtap M (2021). Atrial fibrillation. https://www.ncbi.nlm.nih.gov/books/NBK526072/.

[2] Lane DA, Dagres N, Dan GA (2019). Antithrombotic treatment in patients with atrial fibrillation and acute coronary syndromes: results of the European Heart Rhythm Association survey. Europace.

[3] Steg PG, Bhatt DL (2017). Viewpoint: a proposal for a simple algorithm for managing oral anticoagulation and antiplatelet therapy in patients with non-valvular atrial fibrillation and coronary stents. Eur Heart J Acute Cardiovasc Care.

[4] January CT, Wann LS, Calkins H (2019). 2019 AHA/ACC/HRS focused update of the 2014 AHA/ACC/HRS guideline for the management of patients with atrial fibrillation: a report of the American College of Cardiology/American Heart Association Task Force on Clinical Practice Guidelines and the Heart Rhythm Society. J Am Coll Cardiol.

[5] Amsterdam EA, Wenger NK, Brindis RG (2014). 2014 AHA/ACC guideline for the management of patients with non-ST-elevation acute coronary syndromes: a report of the American College of Cardiology/American Heart Association Task Force on Practice Guidelines. J Am Coll Cardiol.

[6] O'Gara PT, Kushner FG, Ascheim DD (2013). 2013 ACCF/AHA guideline for the management of ST-elevation myocardial infarction: a report of the American College of Cardiology Foundation/American Heart Association Task Force on Practice Guidelines. J Am Coll Cardiol.

[7] Lopes RD, Heizer G, Aronson R (2019). Antithrombotic therapy after acute coronary syndrome or PCI in atrial fibrillation. N Engl J Med.

[8] Heidbuchel H, Verhamme P, Alings M (2015). Updated European Heart Rhythm Association Practical Guide on the use of non-vitamin K antagonist anticoagulants in patients with non-valvular atrial fibrillation. Europace.

[9] Deharo P, Cuisset T (2020). Optimal duration of dual antiplatelet therapy post percutaneous coronary intervention in acute coronary syndrome. Trends Cardiovasc Med.

[10] Eyileten C, Postula M, Jakubik D (2020). Non-vitamin K oral anticoagulants (NOAC) versus vitamin K antagonists (VKA) for atrial fibrillation with elective or urgent percutaneous coronary intervention: a meta-analysis with a particular focus on combination type. J Clin Med.

[11] Hansen ML, Sørensen R, Clausen MT (2010). Risk of bleeding with single, dual, or triple therapy with warfarin, aspirin, and clopidogrel in patients with atrial fibrillation. Arch Intern Med.

[12] Lopes RD, Rao M, Simon DN (2016). Triple vs dual antithrombotic therapy in patients with atrial fibrillation and coronary artery disease. Am J Med.

[13] Sørensen R, Hansen ML, Abildstrom SZ (2009). Risk of bleeding in patients with acute myocardial infarction treated with different combinations of aspirin, clopidogrel, and vitamin K antagonists in Denmark: a retrospective analysis of nationwide registry data. Lancet.

[14] Lopes RD, Vora AN, Liaw D (2018). An open-Label, 2 × 2 factorial, randomized controlled trial to evaluate the safety of apixaban vs. vitamin K antagonist and aspirin vs. placebo in patients with atrial fibrillation and acute coronary syndrome and/or percutaneous coronary intervention: rationale and design of the AUGUSTUS trial. Am Heart J.

[15] Gibson CM, Mehran R, Bode C (2016). Prevention of bleeding in patients with atrial fibrillation undergoing PCI. N Engl J Med.

[16] Cannon CP, Bhatt DL, Oldgren J (2017). Dual antithrombotic therapy with dabigatran after PCI in atrial fibrillation. N Engl J Med.

[17] Eisenberg JM (2002). Physician utilization the state of research about physicians' practice patterns. Medical care.

[18] (2021). Cardiology ACo. Global Anticoagulation Therapy Patterns Vary: Findings from TREAT-RISK. http://www.acc.org/latest-in-cardiology/articles/2012/10/19/16/38/global-anticoagulation-therapy-patterns-vary.

[19] Kumbhani DJ, Cannon CP, Beavers CJ (2021). 2020 ACC expert consensus decision pathway for anticoagulant and antiplatelet therapy in patients with atrial fibrillation or venous thromboembolism undergoing percutaneous coronary intervention or with atherosclerotic cardiovascular disease: a report of the American College of Cardiology Solution Set Oversight Committee. J Am Coll Cardiol.

[20] Potpara TS, Boriani G, Lip GY (2017). Evaluating adherence to non-vitamin-K antagonist oral anticoagulants in post-approval observational studies of patients with atrial fibrillation. Curr Med Res Opin.

[21] Wilke T, Bauer S, Mueller S, Kohlmann T, Bauersachs R (2017). Patient preferences for oral anticoagulation therapy in atrial fibrillation: a systematic literature review. Patient.

[22] Sørensen R, Jamie Nielsen B, Langtved Pallisgaard J, Ji-Young Lee C, Torp-Pedersen C (2017). Adherence with oral anticoagulation in non-valvular atrial fibrillation: a comparison of vitamin K antagonists and non-vitamin K antagonists. Eur Heart J Cardiovasc Pharmacother.

[23] Fei Y, Lam CK, Cheung BM (2020). Efficacy and safety of newer P2Y(12) inhibitors for acute coronary syndrome: a network meta-analysis. Sci Rep.

[24] Steffel J, Verhamme P, Potpara TS (2018). The 2018 European Heart Rhythm Association Practical Guide on the use of non-vitamin K antagonist oral anticoagulants in patients with atrial fibrillation. Eur Heart J.

[25] Lip GY, Collet JP, Haude M (2019). 2018 Joint European consensus document on the management of antithrombotic therapy in atrial fibrillation patients presenting with acute coronary syndrome and/or undergoing percutaneous cardiovascular interventions: a joint consensus document of the European Heart Rhythm Association (EHRA), European Society of Cardiology Working Group on Thrombosis, European Association of Percutaneous Cardiovascular Interventions (EAPCI), and European Association of Acute Cardiac Care (ACCA) endorsed by the Heart Rhythm Society (HRS), Asia-Pacific Heart Rhythm Society (APHRS), Latin America Heart Rhythm Society (LAHRS), and Cardiac Arrhythmia Society of Southern Africa (CASSA). Europace.

[26] Valgimigli M, Bueno H, Byrne RA (2018). 2017 ESC focused update on dual antiplatelet therapy in coronary artery disease developed in collaboration with EACTS. Eur J Cardiothorac Surg.

[27] Čihák R, Haman L, Táborský M (2016). 2016 ESC Guidelines for the management of atrial fibrillation developed in collaboration with EACTS. Cor et vasa.

[28] Angiolillo DJ, Bhatt DL, Cannon CP (2021). Antithrombotic therapy in patients with atrial fibrillation treated with oral anticoagulation undergoing percutaneous coronary intervention: a North American perspective: 2021 update. Circulation.

[29] Pradhan A, Bhandari M, Vishwakarma P, Sethi R (2020). Novel dual therapy: a paradigm shift in anticoagulation in patients of atrial fibrillation undergoing percutaneous coronary intervention. TH Open.

[30] Bauer KA (2013). Pros and cons of new oral anticoagulants. Hematology Am Soc Hematol Educ Program.

[31] Hicks T, Stewart F, Eisinga A (2016). NOACs versus warfarin for stroke prevention in patients with AF: a systematic review and meta-analysis. Open Heart.

[32] Lemesle G, Ducrocq G, Elbez Y (2017). Vitamin K antagonists with or without long-term antiplatelet therapy in outpatients with stable coronary artery disease and atrial fibrillation: association with ischemic and bleeding events. Clin Cardiol.

[33] Mekaj YH, Mekaj AY, Duci SB, Miftari EI (2015). New oral anticoagulants: their advantages and disadvantages compared with vitamin K antagonists in the prevention and treatment of patients with thromboembolic events. Ther Clin Risk Manag.

[34] Alexander JH, Lopes RD, Thomas L (2014). Apixaban vs. warfarin with concomitant aspirin in patients with atrial fibrillation: insights from the ARISTOTLE trial. Eur Heart J.

[35] Fralick M, Colacci M, Schneeweiss S, Huybrechts KF, Lin KJ, Gagne JJ (2020). Effectiveness and safety of apixaban compared with rivaroxaban for patients with atrial fibrillation in routine practice: a cohort study. Ann Intern Med.

[36] Rutherford OW, Jonasson C, Ghanima W, Söderdahl F, Halvorsen S (2020). Comparison of dabigatran, rivaroxaban, and apixaban for effectiveness and safety in atrial fibrillation: a nationwide cohort study. Eur Heart J Cardiovasc Pharmacother.

